# Surgical efficacy and survival prediction of patients with unspecified malignant bone tumors

**DOI:** 10.1186/s12885-022-10153-x

**Published:** 2022-10-20

**Authors:** Shaohui He, Runyi Jiang, Haitao Sun, Jian Yang, Chen Ye, Weibo Liu, Xinghai Yang, Xiaopan Cai, Jianru Xiao

**Affiliations:** 1 Spinal Tumor Center, Department of Orthopaedic Oncology, No.905 Hospital of PLA Navy, Changzheng Hospital, The Second Military Medical University, 415 Fengyang Road, Shanghai, 200003 China; 2 Department of Spine Surgery, Central Hospital of Qingdao, 127 Siliu south Road, Shandong Province, Qingdao, 266042 China

**Keywords:** Unspecific malignant bone tumor, Surgery, Nomogram, Propensity score matching, Prognosis

## Abstract

**Background:**

The surgical efficacy and prognostic outcomes of patients with unspecific malignant bone tumors (UMBTs) remain unclear. The study is to address: 1) What are the clinicopathological features and prognostic determinants for patients with UMBTs? 2) Can a nomogram be developed for clinicians to predict the short and long-term outcomes for individuals with UMBTs? 3) Does surgery improve outcomes for UMBT patients who received radiotherapy or chemotherapy after balancing the confounding bias?

**Methods:**

400 UMBT patients were filtrated from the Surveillance, Epidemiology, and End Results database to assess the clinicopathological features, treatments, and factors affecting prognosis. The optimal cutoff values of continuous variables were identified by the x-tile software. Kaplan-Meier method and multivariate Cox proportional hazard modeling were performed to evaluate the independent prognostic factors. Nomogram was further developed by using R software with rms package. The surgical efficacy was further assessed for patients receiving radiotherapy or chemotherapy after performing propensity score matching.

**Results:**

The enrolled cohort included 195 (48.8%) female and 205 (51.2%) male patients. The 2- and 5-year cancer-specific survival (CSS) and overall survival (OS) rate were 58.2 ± 3.0%, 46.8 ± 3.2%, and 46.5 ± 2.6%, 34.4 ± 2.5%, respectively. Nomogram was finally developed for CSS and OS according to the identified independent factors: age, tumor extent, primary tumor surgery, tumor size, and pathology grade. For UMBT patients who received radiotherapy or chemotherapy, surgical intervention was associated with better CSS (pr = 0.003, pc = 0.002) and OS (pr = 0.035, pc = 0.002), respectively.

**Conclusions:**

Nomogram was developed for individual UMBT patient to predict short and long-term CSS and OS rate, and more external patient cohorts are warranted for validation. Surgery improves outcomes for UMBT patients who received either radiotherapy or chemotherapy.

**Supplementary Information:**

The online version contains supplementary material available at 10.1186/s12885-022-10153-x.

## Background

The unspecific malignant bone tumors (UMBTs) are a group of bone tumors in addition to the specific bone tumors (e.g. osteosarcoma, chondrosarcoma, Ewing sarcoma, etc). The definition of UMBTs are provided by the International Classification of Childhood Cancer, Third Edition (ICCC-3) based on the International Classification of Diseases for Oncology, Third Edition (ICD-O-3) [1]. The UMBT is relatively rare, which only accounts for 1.2% among all the bone malignancies [2]. Compared to the histology-specific bone tumors, limited information was published regarding the clinical characteristics, treatments, and prognosis of UMBTs.

The Surveillance, Epidemiology, and End Results (SEER) database (http://seer.cancer.gov) provides a nationwide, population-based patients to help study the epidemic features, efficacy of treatments, and prognostic outcomes of different cancer patients [3–5]. Therefore, we utilized the SEER database to possibly address the following clinical questions: 1) What are the clinicopathological features and prognostic determinants for patients with UMBTs? 2) Can a nomogram be developed for clinicians to predict the preliminary and long-term outcomes for individuals with UMBTs? 3) Does surgery improve outcomes for UMBT patients who received radiotherapy or chemotherapy after balancing the data bias?

## Methods

### Study population

UMBTs were defined as a group of bone tumors in addition to osteosarcoma, chondrosarcoma, Ewing sarcoma and other specific bone tumors, which were provided by the ICCC-3 and encoded in the SEER database. By using the SEER*Stat software (Version 8.3.5, National Cancer Institute, Bethesda, MD, USA), the clinical information of UMBT patients from 1973 to 2016 was obtained from SEER database, which covers 30% US population [6, 7]. According to the ICD-O-3, the codes for selected histologic types were 8000–8005, 8800–8801, and 8803–8805, and the primary site codes were C40.0–40.3, C40.9–41.4, and C41.8–41.9. The anatomic site record was “bone and joints”. This study was a retrospective design with duration of over 40 years (1973–2016), and all the enrolled patients were from the USA population.

### Inclusion and exclusion

In our study, the inclusion criteria were as follows: 1) Confirmed diagnosis of UMBT with positive histology; 2) the diagnosis was acquired at a living status; 3) active follow up was confirmed for each patient; 4) definite survival months. While the enrolled patients were further excluded for at least one of the following reasons: 1) incomplete information of tumor sites; 2) lack of surgical information; 3) patients under 18 years old; 4) the survival month was 0; 5) number of patients from Alaska < 11(please note we did not give the exact number to protect patient privacy). Notably, according to the provided information of the SEER database, tumor sites were divided into the following three subgroups for further analysis: limbs (including upper and lower limbs, and associated joints), and the spine (including vertebral column/pelvis, sacrum, coccyx, and associated joints), and others (including mandible/skull, face and associated joints/rib, sternum, clavicle and associated joints).

### Baseline information

**Fig. 1:**
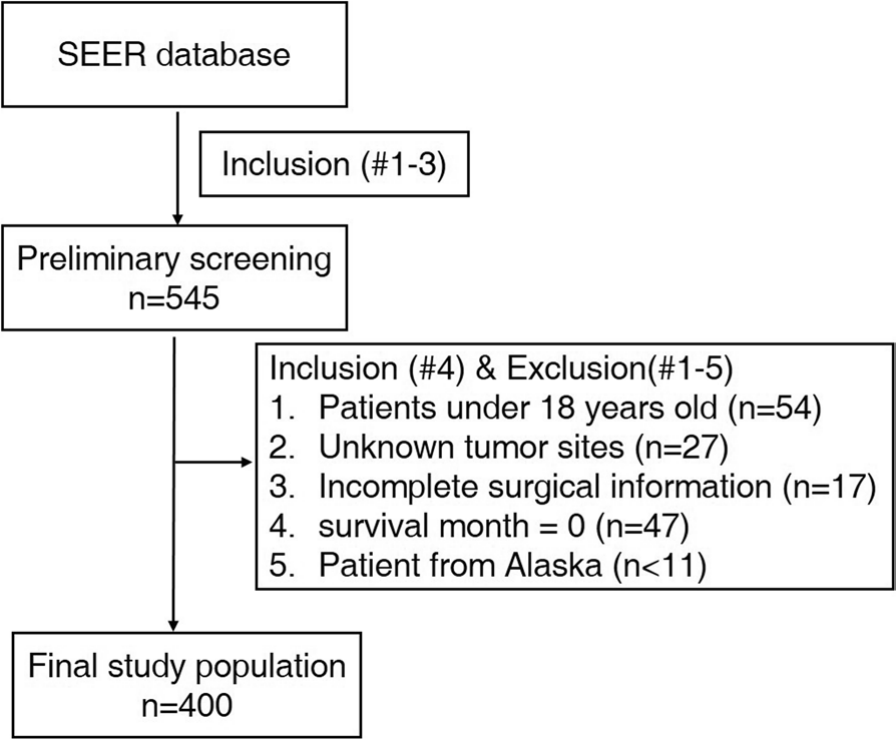
The flowchart of step-by-step screening process of eligible patients (*n* = 400)

The collected clinicopathological factors included: age, race, sex, year of diagnosis, region (southwest/pacific coast/northern plains/east), primary tumor site, pathological grade, tumor laterality, tumor extent (localized/regional/distant), primary tumor surgery, radiotherapy and chemotherapy, tumor size, initial tumor diagnosed, hispanic/non-hispanic patients, marital status, and annual family income. The primary endpoints were cancer-specific survival (CSS) and overall survival (OS). CSS was defined as the interval from the first day to the death date due to UMBT or the end of follow-up, while OS was defined as the duration from the first day to the date of all-cause death or the end day of follow-up. Figure 1 showed the flow chart of patients collecting process: in short, 545 UMBT patients were initially screened out from the case information list via SEER*Stat software, then 145 patients were further excluded for the following reasons: Age < 18 years old(*n* = 54), unknown tumor site (*n* = 27), incomplete surgical information (*n* = 17), 0 survival month of follow up (*n* = 7), and the patient from Alaska (*n* = 1) was also removed because of the limited number. Finally, 400 UMBT cases were enrolled in our study for further analysis.

### Statistical analysis

**Fig. 2:**
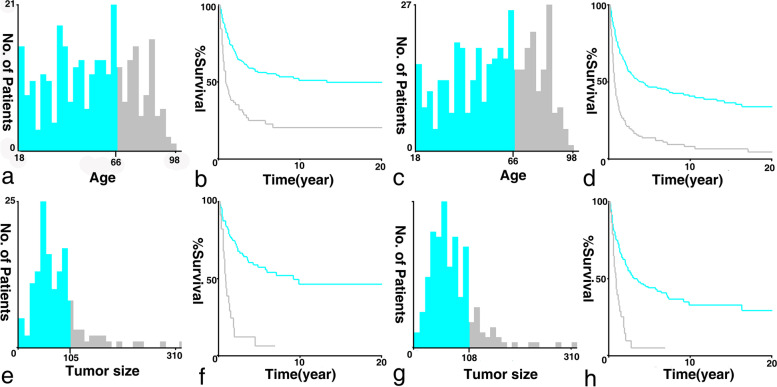
The histogram and survival curves of UMBT patients based on the identified optimal cutoff values. The x-tile histograms and Kaplan-Meier curves were illustrated for cancer-specific and overall survival when (**A-D**) Age cutoff value was 66 years old; **E-H** Tumor size cutoff values were 105 and 108 mm, respectively

The mean with standard deviation (SD) and/or range were used to describe the quantitative data, while counts and percentages for qualitative data. The cutoff values of continuous factors were identified optimally by using the x-tile software (Yale University, New Haven, CT, USA) [8]. Theoretically, the optimal cutoff value was generated based on the highest chi-square values calculated upon the division. Notably, the results showed that the binary cutoff values of CSS and OS were described as follows: 66 years old for age, and 105/108 mm for tumor size, respectively (Fig. 2).

Univariate analysis was then performed by the SPSS 22.0 (SPSS Inc., Chicago, IL, USA) to figure out the potential significant factors by log-rank test, and parameters with *p* < 0.05 were further subjected to multivariate Cox proportional hazard model to identify the independent determinants with estimated hazard ratios. Notably, since the information of patients who did not receive radiotherapy and chemotherapy was incomplete in the SEER database (“None/Unknown” for radiotherapy, and “No/Unknown” for chemotherapy), these two factors were not subjected into survival analysis. Kaplan-Meier curve of independent factors was drawn by the GraphPad Prism 7.0 (GraphPad Software, San Diego, CA, USA).

After identifying independent contributors by multivariate analysis, Nomogram was further developed by using R software (version 3.5.1) with rms package (available at: http://CRAN.R-project.org/package=rms). The developed nomogram can be used to predict the 2 and 5-year CSS and OS, respectively. Harrell’s concordance index (C-index) and calibration curves were generated to assess the predictive value of the nomogram by comparing with the actual proportions.

Besides, the clinical features between surgery and non-surgery groups were assessed by using student *t* and Pearson χ^2^ test or Fisher exact test. Propensity score matching (PSM) method (R with MatchIt package, available at: http://CRAN.R-project.org/package=MatchIt) was utilized to balance the impact caused by the unmatched bias. The matching ratio was designated as 1:1 with caliper of 0.05 for surgery and non-surgery groups. After PSM, the effects of surgical treatments on CSS and OS were further evaluated among patients receiving either radiotherapy or chemotherapy, respectively. The related death rate curves were analyzed and depicted by using the GraphPad Prism 7.0 (GraphPad Software, San Diego, CA, USA), with *p* < 0.05 being regarded statistically significant.

## Results

The baseline features of 400 UMBT patients were summarized in Table S1. The study population consisted of 195 (48.8%) female and 205 (51.2%) male patients, with the average age of 59.6 ± 19.7 and 55.2 ± 20.3 years old, respectively. 194 UMBTs originated from limbs, and 132 cases occurred in mandible/skull, face and associated joints/rib, sternum, clavicle and associated joints, while UMBTs of the spine accounted for 18.5%. Two hundred ninety-two patients had confirmed CSS status, while OS status was obtained from all patients (*n* = 400, 100%). During the mean follow-up of 49.4 ± 76.8 months (range 1–437), the 2, 5-year CSS and OS rate were 58.2 ± 3.0%, 46.8 ± 3.2%, and 46.5 ± 2.6%, 34.4 ± 2.5%, respectively.

**Fig. 3:**
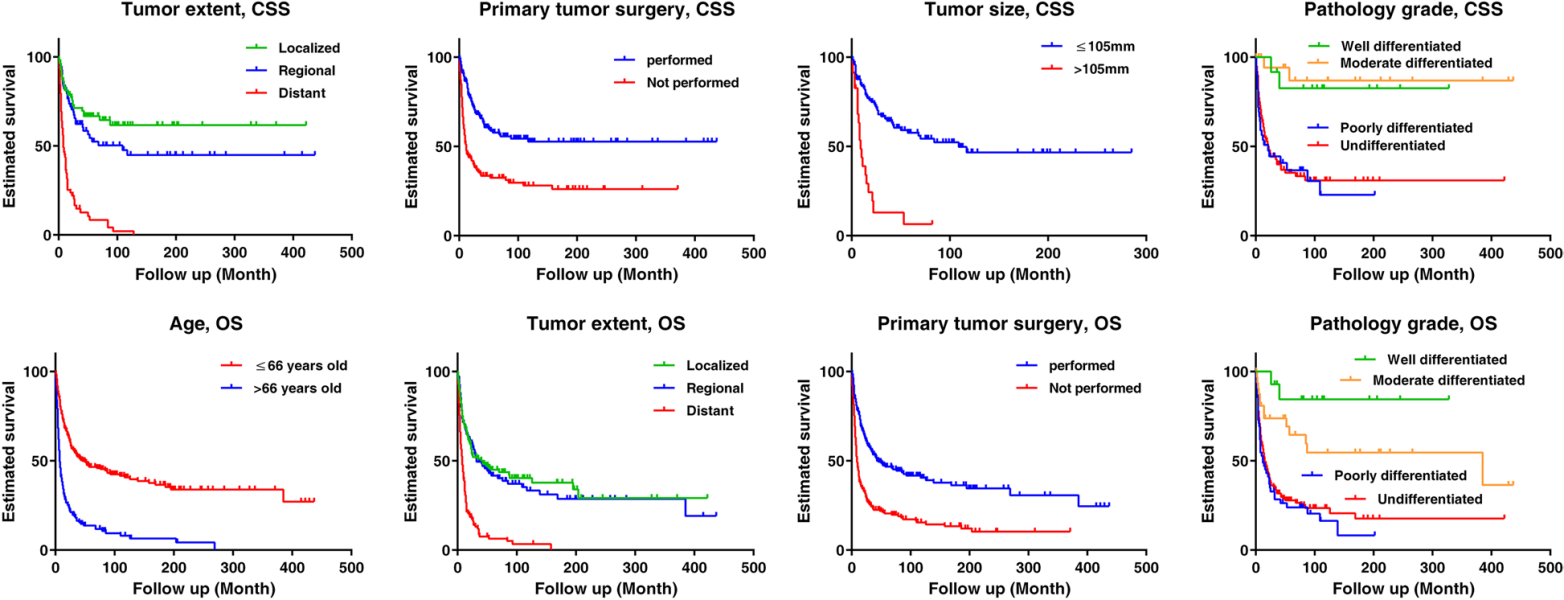
The Kaplan-Meier curves were delineated according to the identified independent prognostic factors, including (**A-D**) Tumor extent (*p* < 0.001), primary tumor surgery (*p* < 0.001), tumor size (*p* < 0.001) and pathology grade (*p* < 0.001) for CSS; **E-H** Age, tumor extent, primary tumor surgery (*p* < 0.001), and pathology grade (*p* < 0.001) for OS. CSS = Cancer-specific survival; OS=Overall survival

The univariate analysis (Table 1) indicated that the potential significant factors for CSS and OS were age (*p* < 0.001 for both), diagnosis year (*p* = 0.048 & *p* = 0.011), tumor site (*p* < 0.001 for both), tumor extent (*p* < 0.001 for both), tumor size (*p* < 0.001 for both), primary tumor surgery (*p* < 0.001 for both), pathology grade (*p* < 0.001 for both), first tumor diagnosed (*p* < 0.001 for OS), marital status (*p* < 0.001 for both). The related 5-year survival rates for subgroups were also shown in Table 1. The potential factors were then subjected to multivariate Cox proportional hazard model, and the results (Table 2) revealed tumor extent (*p* < 0.001), primary tumor surgery (*p* = 0.018), tumor size (*p* = 0.027), and pathology grade (*p* = 0.005) were independently associated with CSS. While the contributors for OS were age (*p* = 0.001), tumor extent (*p* = 0.002), primary tumor surgery (*p* < 0.001), and pathology grade (*p* = 0.010). The Kaplan-Meier curves regarding the independent factors were illustrated in Fig. 3.

**Table 1:** Univariate analysis of preoperative factors and surgery affecting cancer-specific and overall survival

Factors	Cancer-specific survival			Overall survival		
	n	5-year Survival rate (%, mean ± SE)	***p value***	n	5-year Survival rate (%, mean ± SE)	***p value***
Age:18–66/> 66 yrs	201/91	56.1 ± 3.8/24.8 ± 5.2	< 0.001	254/146	46.5 ± 3.4/13.7 ± 3.0	< 0.001
Race: Black/white/others^a^	37/235/13	56.5 ± 8.6/44.7 ± 3.6 /27.7 ± 15.0	0.532	48/323/23	45.7 ± 7.8/32.7 ± 2.7 /17.2 ± 8.9	0.408
Gender: Female/male	138/154	46.6 ± 4.5/46.7 ± 4.5	0.434	195/205	35.5 ± 3.6/33.3 ± 3.5	0.895
Diagnosis year: ≤2000/> 2000	77/215	53.0 ± 5.8/44.2 ± 3.9	0.048	94/306	43.6 ± 5.1/31.1 ± 2.9	0.011
Region: Southeast/pacific coast/ northern plains/east	28/118/ 28/118	23.6 ± 11.9/42.3 ± 5.2/ 36.3 ± 9.4/53.7 ± 4.9	0.533	34/176/ 38/152	32.5 ± 9.2/31.2 ± 3.8/ 25.2 ± 7.2/40.3 ± 4.1	0.516
Site: Limbs/spine/others^b^	161/46/85	49.1 ± 4.3/67.3 ± 7.5 /31.2 ± 5.6	< 0.001	194/74/132	40.3 ± 3.7/48.5 ± 6.3 /17.8 ± 3.6	< 0.001
Tumor laterality: Left/right	111/95	51.6 ± 5.1/40.6 ± 5.6	0.145	139/139	41.2 ± 4.3/30.3 ± 4.2	0.070
Tumor extent: Localized/regional/distant	84/84/79	66.7 ± 5.5/52.4 ± 6.4 /8.5 ± 3.6	< 0.001	123/112/108	44.9 ± 4.7/41.6 ± 5.2 /6.3 ± 2.5	< 0.001
Tumor size: ≤105(108)/105(108) mm	116/23	57.6 ± 5.2/6.5 ± 6.0	< 0.001	167/29	44.0 ± 4.2/4.8 ± 4.5	< 0.001
Primary tumor surgery: Yes/no	164/128	57.7 ± 4.3/32.4 ± 4.4	< 0.001	213/187	46.7 ± 3.7/20.5 ± 3.1	< 0.001
Pathology grade: I/II/III/IV	12/21/39/100	82.5 ± 11.3/86.9 ± 8.7/ 36.7 ± 8.4/35.3 ± 5.3	< 0.001	14/34/55/137	84.4 ± 10.2/64.5 ± 9.3 /23.8 ± 6.1/27.8 ± 4.1	< 0.001
First tumor diagnosed: Yes/no	292/0	\	\	293/107	40.7 ± 3.0/16.7 ± 4.0	< 0.001
Annual income aggregate level: Low/High (Cutoff: 75,000USD)	162/130	43.5 ± 4.3/50.7 ± 4.7	0.185	210/190	32.4 ± 3.5/36.4 ± 3.6	0.338
Hispanic: Yes/no	43/249	42.2 ± 7.9/47.7 ± 3.5	0.422	52/348	40.0 ± 7.2/33.7 ± 2.7	0.516
Marital status: Single/married/others^c^	135/66/68	48.1.1 ± 4.8/51.0 ± 6.6 /32.8 ± 6.1	< 0.001	191/82/97	34.2 ± 3.7/43.8 ± 5.8 /21.9 ± 4.4	< 0.001

*SE* standard error, *yrs* years old, *USD* US dollar

^a^including: American Indian/AK Native, Asian/Pacific Islander

^b^including: mandible/skull, face and associated joints/rib, sternum, clavicle and associated joints

^c^including: divorced, separated, widowed

**Table 2:** Multivariate analysis of clinicopathological factors affecting cancer-specific and overall survival

Factors	Cancer-specific survival		Overall survival	
	HR (95% CI)	***p*** value	HR (95% CI)	***p*** value
Age	**\**	**\**	2.255(1.415–3.593)	0.001
Tumor extent	3.186(1.827–5.556)	< 0.001	1.652(1.194–2.285)	0.002
Primary tumor surgery	0.438(0.221–0.869)	0.018	0.309(0.191–0.501)	< 0.001
Tumor size	2.121(1.087–4.139)	0.027	\	\
Pathology grade	2.028(1.237–3.325)	0.005	1.526(1.106–2.105)	0.010

*HR* hazard ratio, *CI* confidence interval

**Fig. 4:**
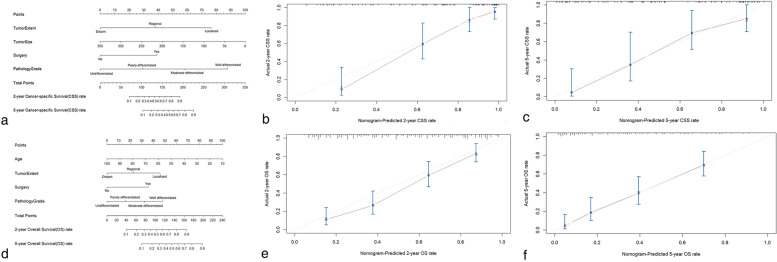
The graphs showed nomograms and related calibration curves for 2, 5-year (**A-C**) cancer-specific survival; **D-F** overall survival, respectively. For example, when a 60-year-old patient (CSS points = N/A, OS points = 45) with regional (CSS points = 38, OS points = 23), poorly differentiated (CSS points = 30, OS points = 18) UMBT (tumor size = 100 mm [CSS points = 72, OS points = N/A], no previous primary tumor surgery [CSS points = 0, OS points = 0]) were presented to the institution, total points of 140 and 86 were assessed on this patient for CSS and OS estimation, respectively. The estimated 2, 5-year CSS and OS rates were 58, 32 and 35%, 16%, respectively

The nomograms were further developed to predict CSS and OS rate for individual UMBT patient by analyzing the above factors (Fig. 4). The developed nomograms can be used conveniently in the clinical practice. For example, when a 60-year-old patient with regional, poorly differentiated UMBT (tumor size = 100 mm, no previous primary tumor surgery) were presented to the institution, total points of 150 and 86 were assessed on this patient for CSS and OS, respectively. The estimated 2, 5-year CSS and OS rates were 65, 38 and 35%, 16%, respectively. The C-index of CSS and OS prediction for UMBTs were 0.829 ± 0.054 and 0.752 ± 0.043, respectively, and the calibration plots (Fig. 4b-c & e-f) showed favorable consistence between nomogram-based prediction and actual outcomes of 2, 5-year CSS and OS for UMBT patients, respectively.

**Fig. 5:**
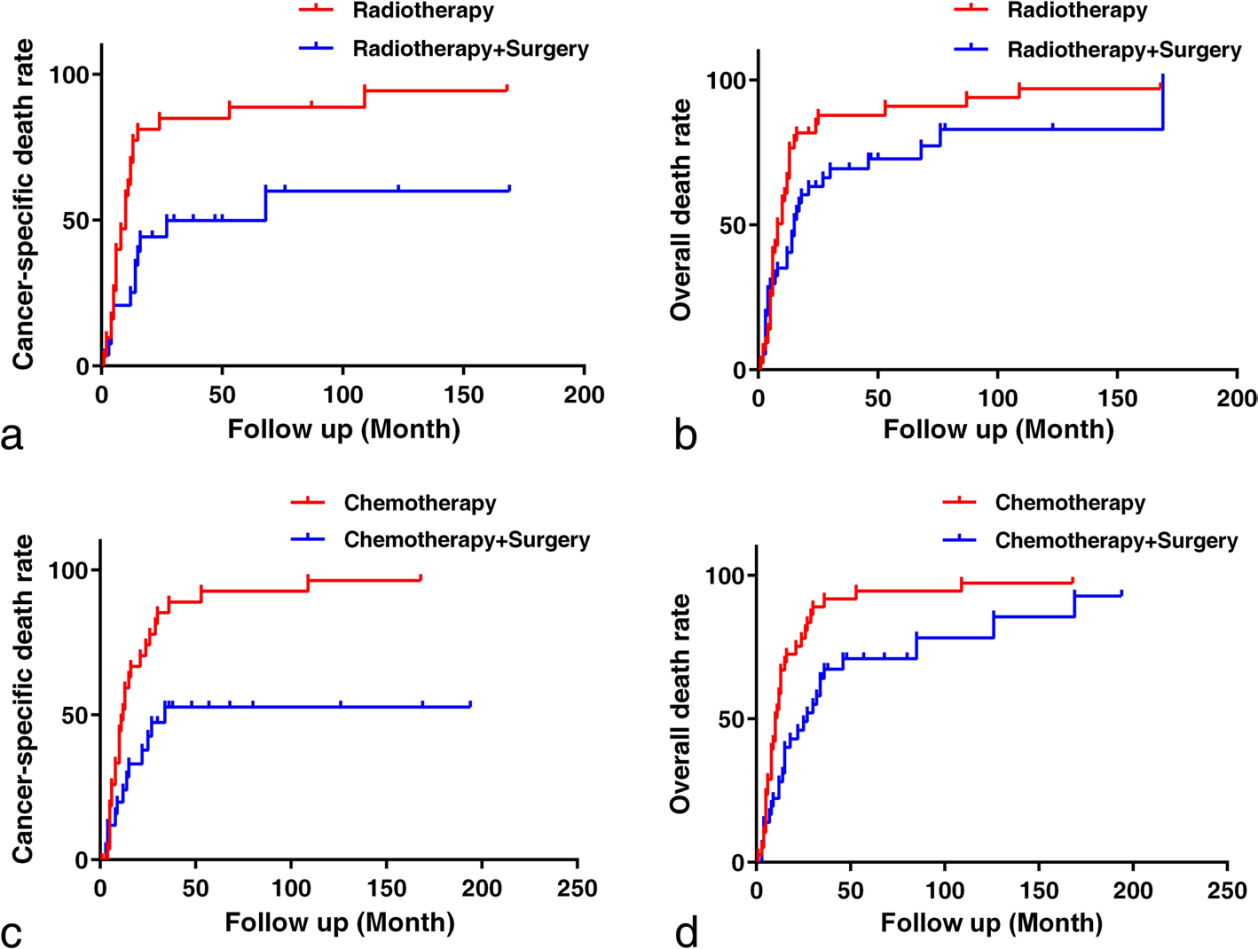
The graphs revealed Kaplan-Meier curves between the following groups (data from the propensity score sample): **A-B** surgery + radiotherapy vs. radiotherapy for cancer-specific (*p* = 0.003) and overall death rate (*p* = 0.002); **C-D** surgery + chemotherapy vs. chemotherapy for cancer-specific (*p* = 0.035) and overall death rate (*p* = 0.002)

Meanwhile, the effect of surgery on UMBT patients who underwent radiation and chemotherapy were further explored after using the PSM method for imbalance mitigation. The distributions of propensity score after performing PSM were delineated in Fig. S1. As shown in Table 3, the baseline bias for unmatched patients existed among the following factors: age (*p* < 0.001), sex (*p* = 0.010), tumor site (*p* < 0.001), tumor extent (*p* < 0.001), pathology grade (*p* = 0.041), first tumor diagnosed (*p* = 0.042), marital status (*p* = 0.001), and family income (*p* = 0.001). However, after performing PSM, the bias was mitigated for age (*p* = 0.533), sex (*p* = 0.879), tumor site (*p* = 0.070), pathology grade (*p* = 0.568), first tumor diagnosed (*p* = 0.308), marital status (*p* = 0.520), and family income (*p* = 0.570). One hundred seventy-two cases (86:86) were finally matched to explore the surgical effect for patients who received either radiotherapy or chemotherapy, respectively. According to the results (Fig. 5), compared with patients who only received radiotherapy, surgery plus radiation increased CSS (*p* = 0.003) and OS rates (*p* = 0.035). Likewise, lower cancer-specific (*p* = 0.002) and overall death rates (*p* = 0.002) were observed for patients who underwent combined surgery and chemotherapy.

**Table 3:** Baseline characteristics between surgery and non-surgery groups

Factors	Primary tumor surgery (Unmatched)		***p*** value	Primary tumor surgery (Propensity score matching)		***p*** value
	Yes (***n*** = 213)	No (***n*** = 187)		Yes (***n*** = 86)	No (***n*** = 86)	
Age (yrs, mean ± SD)	52.6 ± 19.5	62.8 ± 19.3	< 0.001	56.3 ± 18.7	58.1 ± 19.3	0.533
Race (n,%)			0.107			0.142
Black	20 (9.4)	28 (15.0)		6 (7.0)	14 (16.3)	
White	181 (85.0)	142 (75.9)		77 (89.5)	68 (79.1)	
Others^a^	10 (4.7)	13 (7.0)		3 (3.5)	4 (4.6)	
Sex (n,%)			0.010			0.879
Female	91 (42.7)	104 (55.6)		41 (47.7)	42 (48.8)	
Male	122 (57.3)	83 (44.4)		45 (52.3)	44 (51.2)	
Diagnosis year (n,%)			0.160			0.578
≤ 2000	56 (26.3)	38 (20.3)		20 (23.3)	17 (19.8)	
> 2000	157 (73.7)	149 (79.7)		66 (76.7)	69 (80.2)	
Region (n,%)			0.935			0.852
Southeast	17 (8.0)	17 (9.1)		6 (6.9)	8 (9.3)	
Pacific coast	96 (45.1)	80 (42.8)		36 (41.9)	39 (45.3)	
Northern plains	19 (8.9)	19 (10.2)		9 (10.5)	9 (10.5)	
East	81 (38.0)	71 (38.1)		35 (40.7)	30 (34.9)	
Tumor site (n,%)			< 0.001			0.070
Limbs	128 (60.1)	66 (35.3)		34 (39.5)	39 (45.3)	
Spine	45 (21.1)	29 (15.5)		23 (26.8)	11 (12.8)	
Others^b^	40 (18.8)	92 (49.2)		29 (33.7)	36 (41.9)	
Tumor laterality (n,%)			0.277			0.097
Left	82 (38.5)	57 (30.8)		27 (31.4)	32 (37.2)	
Right	73 (34.3)	66 (35.3)		25 (29.1)	32 (37.2)	
Tumor extent			< 0.001			0.032
Localized	76 (35.7)	47 (25.1)		21 (24.4)	20 (23.3)	
Regional	84 (39.4)	28 (15.0)		36 (41.9)	20 (23.3)	
Distant	36 (16.9)	72 (38.5)		29 (33.7)	36 (41.9)	
Tumor size (mm, mean ± SD)	71.5 ± 44.9	80.5 ± 46.8	0.204	77.6 ± 52.0	80.1 ± 46.5	0.812
Pathology grade (n,%)			0.041			0.568
I: Well differentiated	11 (51.6)	3 (1.6)		3 (3.5)	1 (1.2)	
II: Moderately differentiated	27 (12.7)	7 (3.7)		8 (9.3)	5 (5.8)	
III: Poorly differentiated	30 (14.1)	25 (13.4)		11 (12.8)	14 (16.3)	
IV: Undifferentiated	79 (37.1)	58 (31.0)		35 (40.7)	35 (40.7)	
First tumor diagnosis (n,%)			0.042			0.308
Yes	165 (77.5)	128 (68.5)		21 (24.4)	27 (31.4)	
No	48 (22.5)	59 (31.6)		65 (75.6)	59 (68.6)	
Hispanic (n,%)			0.813			0.670
No	184 (86.4)	164 (87.7)		74 (86.0)	72 (83.7)	
Yes	29 (13.6)	23 (12.3)		12 (14.0)	14 (16.3)	
Marital status (n,%)			0.001			0.520
Single	111	80		17 (19.8)	17 (19.8)	
Married	53	29		53 (61.6)	47 (54.7)	
Others^c^	38	59		16 (18.6)	22 (25.5)	
Family income (USD, mean ± SD)	80,780 ± 20,320	74,620 ± 19,630	0.002	74,140 ± 19,590	75,830 ± 20,110	0.570

^a^including: American Indian/AK Native, Asian/Pacific Islander

^b^including: mandible/skull, face and associated joints/rib, sternum, clavicle and associated joints

^c^including: divorced, separated, widowed

## Discussion

UMBTs are much less common than the specific bone malignancies, including osteosarcoma, chondrosarcoma, and Ewing sarcoma, etc. To our knowledge, due to the rarity of UMBTs, no study has been reported regarding the clinical features, related treatments, and contributors for survival towards such patients. Meanwhile, the application of nomogram has been validated as an effective mean for clinicians to anticipate prognosis in different malignant bone tumors [6, 9, 10], thus a well-defined nomogram based on the prognostic factors is urgently required to predict survivals for UMBT patients. Moreover, although surgical resection was reported to be effective in treating various specific malignant bone tumors regardless of metastatic status [6, 11–14], the surgical efficacy for UMBTs remains to be elucidated. The SEER database is the most common used clinical database which consists of about 30% of population from 18 registries that represent all regions of US [3]. It contains various sociodemographic and clinicopathological factors for oncologists to analyze the relevance between risks and tumorigenesis [15, 16], and the prognostic factors affecting CSS and OS [17, 18].

In our study, the estimated 5-year CSS rate for non-metastatic (localized and regional status) and metastatic UMBT patients were 53.2 ± 4.7% and 6.4 ± 3.3%, which were much worse than those for relevant osteosarcoma (71.8 and 30.4%) [19] and Ewing sarcoma patients (72.3 and 35.7%) [20]. Because of the dismal survival outcomes, it is essential to identify the prognostic determinants and effective therapeutics by assessing the large-scale, population-based UMBT patients from SEER database. We firstly identified the survival contributors for UMBTs, including age (only for OS), tumor extent, primary tumor surgery, tumor size (only for CSS), and pathology grade, the nomogram was then developed for UMBTs patients based on the independent factors. The C-index (0.829 ± 0.054 and 0.752 ± 0.043) and calibration curves (Fig. 4) indicated possible feasibilities in predicting the individual CSS and OS rate in the clinical practice.

These clinicopathological factors were also confirmed in assessing other specific malignant bone tumors [6, 10, 13]. Although this is the first-reported nomogram for UMBTs, limitation does exist as follows: 1) The information of radiotherapy and chemotherapy is incomplete in the database, we could only obtain the definite number of patients receiving radiation or chemotherapy, but failed to get the definite numbers for those who did not underwent radiotherapy or chemotherapy due to the “None/Unknown” labeling. Therefore, the prognostic role of radiotherapy and chemotherapy cannot be evaluated completely and independently. 2) Despite favorable C-index and calibration curves, external cohorts are necessary for nomogram validation. 3) As noted in the previous study [6], the SEER database is lack of some well-defined prognostic factors, such as pathologic fracture and other imagelogical parameters. 4) Last but not the least, our study excluded 145 UMBT patients because of age < 18 years old and incomplete information, and nearly 1/4 of the rest patients failed to provide definite cancer-specific status, which may pose an impact on the nomogram development.

In consideration of importance of both surgical and non-surgical treatments, by accessing the available information from SEER database, we further studied the role of primary tumor resection among UMBT patients who received radiotherapy or chemotherapy. PSM was regarded as a useful method to generate similar cases between comparative groups after maximally balancing the bias [21]. Our findings indicated surgery could bring a survival advantage for UMBTs who received radiotherapy or chemotherapy. In other words, the combination of primary tumor resection and radiotherapy/chemotherapy is superior to sole adjuvant therapy for both CSS and OS status. However, we admitted that, due to the limited information of SEER database, we failed to clearly separate “before surgery” from “after surgery” regarding the administration of radiation or chemotherapy. From our own experience, we routinely collected the detailed medical history and assessed imagelogical findings of such patients, including age, tumor site, tumor size, previous treatments, etc. Positron Emission Tomography-Computed Tomography was applied to detect the potential distant metastasis if necessary. Surgical strategy was then conducted to remove the tumor integrally, and chemotherapy (general regimen for sarcoma) was applied if the pathology report indicated high degree of malignancy or metastatic risk. Besides, if the tumor size was large enough, which put high risk for operation, we might conduct radiotherapy before surgery. Last but not least, the rigorous postoperative follow-up was applied to monitor the patient’s recovery, usually monthly for the first 3 months and at 3-month intervals for the next 12 months on the outpatient basis.

## Conclusions

The nomograms are preliminarily developed for UMBT patients after identify independent prognostic factors, and it warrants more external large-scale cohort to validate their feasibilities. The combination of surgery and radiotherapy/chemotherapy for UMBTs is associated with better CSS and OS than those who solely received radiotherapy/chemotherapy.

## Supplementary Information

**Additional file 1.**

**Additional file 2: Fig. S1.** The graphs showed the distributions of propensity score after performing PSM in bubble and bar forms. PSM = Propensity score matching.

**Additional file 3: Table S1.** Baseline characteristics of patients with UMBT (*n* = 400).

## Data Availability

All data generated or analyzed during this study are included as supplemental material (Additional file 1) in this published article.

## Abbreviations

UMBT: Unspecific malignant bone tumor
SEER: The Surveillance, Epidemiology, and End Results
CSS: Cancer-specific survival
OS: Overall survival
C-index: Concordance index
PSM: Propensity score matching
HR: Hazard ratio
CI: Confidence interval
