# Supplementary figures and images for: Surgical efficacy and survival prediction of patients with unspecified malignant bone tumors
Source: BMC Cancer. 2022 Oct 20;22:1078. doi: 10.1186/s12885-022-10153-x (PMC9583561; doi:10.1186/s12885-022-10153-x)

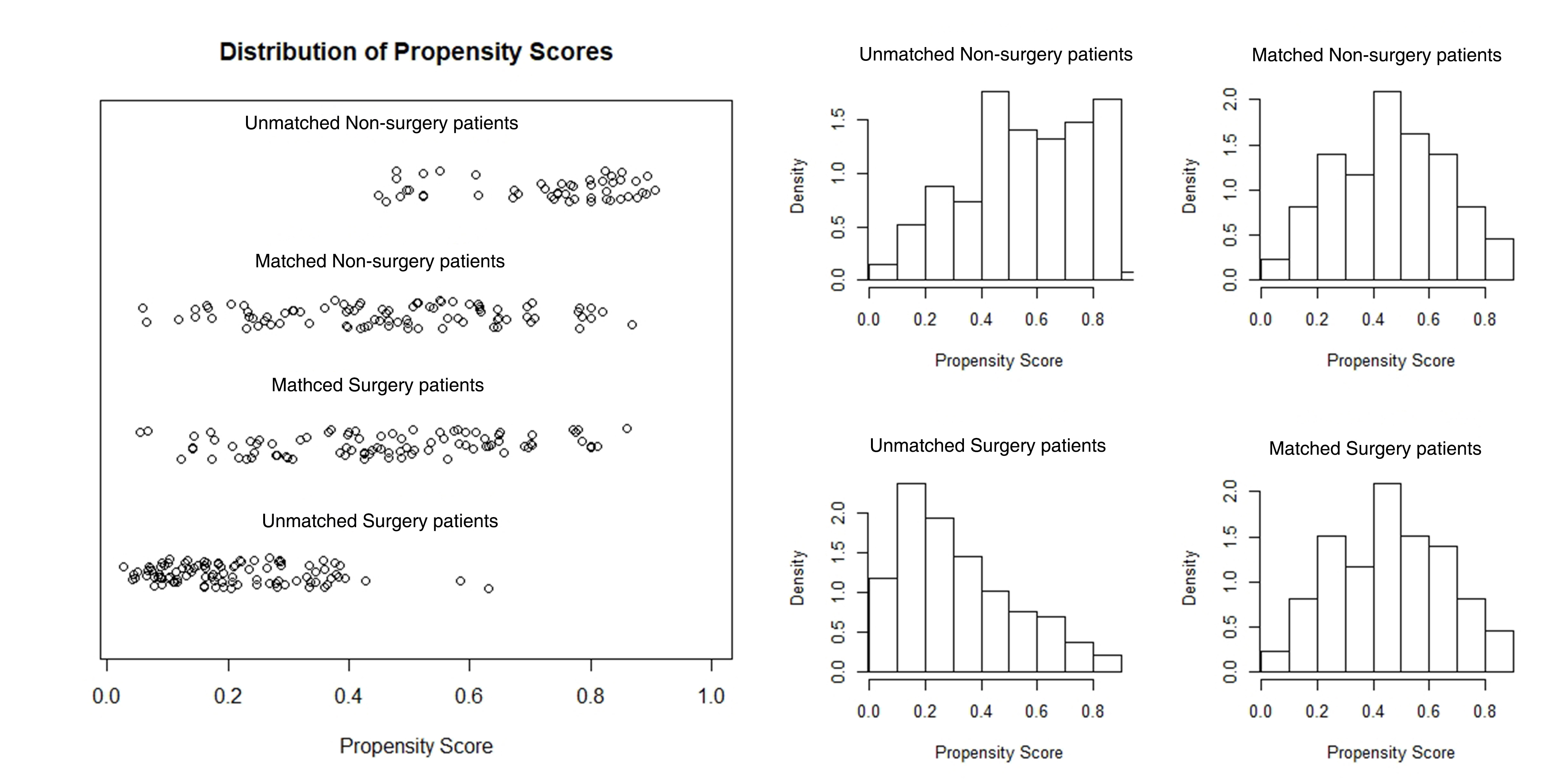

Supplement: Supplementary file 2 — Additional file 2: Fig. S1. The graphs showed the distributions of propensity score after performing PSM in bubble and bar forms. PSM = Propensity score matching. [file 12885_2022_10153_MOESM2_ESM.jpg]
