# Supplementary material for: Surgical efficacy and survival prediction of patients with unspecified malignant bone tumors
Source: BMC Cancer. 2022 Oct 20;22:1078. doi: 10.1186/s12885-022-10153-x (PMC9583561; doi:10.1186/s12885-022-10153-x)
Supplement: Supplementary file 3 — Additional file 3: Table S1. Baseline characteristics of patients with UMBT (n = 400). [file 12885_2022_10153_MOESM3_ESM.docx]

**Table S1 Baseline characteristics of patients with UMBT (n=400)**

| **Age (yrs, Mean±SD）** | 57.4±20.1 | **Tumor size (mm, mean±SD)** | 74.3±45.6 |
| --- | --- | --- | --- |
| **Race (n,%)** |  | **Marital status (n,%)** |  |
| **Black** | 48 (12.0) | **Single** | 191 (47.8) |
| **White** | 323 (80.8) | **Married** | 82 (20.5) |
| **Others*** | 28 (7.0) | **Divorced, separated, widowed** | 97 (24.2) |
| **Unknown** | 6 (1.5) | **Unknown** | 30 (7.5) |
| **Sex (n,%)** |  | **First tumor diagnosed (n,%)** |  |
| **Female** | 195 (48.8) | **Yes** | 293 (73.3) |
| **Male** | 205 (51.2) | **No** | 107 (26.7) |
| **Diagnosis year (n,%)** |  | **Annual family income (USD, n,%)** |  |
| **≤2000** | 94 (23.5) | **≤75,000** | 210 (52.5) |
| **>2000** | 306 (76.5) | **>75,000** | 190 (47.5) |
| **Region (n,%)** |  | **Primary tumor surgery (n,%)** |  |
| **Southeast** | 34 (8.5) | **Yes** | 213 (53.3) |
| **Pacific coast** | 176 (44.0) | **No** | 187 (46.7) |
| **Northern plains** | 38 (9.5) | **Radiotherapy (n,%)** |  |
| **East** | 152 (38.0) | **Yes** | 161 (40.3) |
| **Tumor site (n,%)** |  | **No/Unknown** | 239 (59.7) |
| **Limbs** | 194 (48.5) | **Chemotherapy (n,%)** |  |
| **Spine** | 74 (18.5) | **Yes** | 141 (35.3) |
| **Others**** | 132 (33.0) | **No/Unknown** | 259 (64.7) |
| **Tumor laterality (n,%)** |  | **Combined surgery and radiation** |  |
| **Left** | 139 (34.8) | **Yes** | 76 (19.0) |
| **Right** | 139 (34.8) | **No/Unknown** | 324 (81.0) |
| **Tumor extent** |  | **CSS (n,%)** |  |
| **Localized** | 123 (30.8) | **Alive** | 146 (36.5) |
| **Regional** | 112 (28.0) | **DOD** | 146 (36.5) |
| **Distant** | 108 (27.0) | **Unknown** | 108 (27.0) |
| **Unstaged/Unknown** | 57 (14.2) | **2-year CSS rate (%,mean±SD)** | 58.2±3.0 |
| **Pathology grade (n,%)** |  | **5-year CSS rate (%,mean±SD)** | 46.8±3.2 |
| **I: Well differentiated** | 14 (3.5) | **OS (n,%)** |  |
| **II: Moderately differentiated** | 34 (8.5) | **Alive** | 131 (32.8) |
| **III: Poorly differentiated** | 55 (13.8) | **Dead (all causes)** | 269 (67.2) |
| **IV: Undifferentiated** | 137 (34.2) | **2-year OS rate (%,mean±SD)** | 46.5±2.6 |
| **Unknown** | 160 (40.0) | **5-year OS rate (%,mean±SD)** | 34.4±2.5 |
| **Hispanic (n,%)** |  | **Follow up (month, mean±SD)** | 49.4±76.8 |
| **Yes** | 52 (13.0) |  |  |
| **No** | 348 (87.0) |  |  |

UMBT: unspecified malignant bone tumors; SD: standard deviation; CSS: cancer-specific survival; DOD: died of disease; OS: overall survival.

**^*^** including: American Indian/AK Native, Asian/Pacific Islander

^**^ including: mandible/skull, face and associated joints/rib, sternum, clavicle and associated joints
